# Optimization of a large-scale gene disruption protocol in *Dictyostelium *and analysis of conserved genes of unknown function

**DOI:** 10.1186/1471-2180-6-75

**Published:** 2006-08-31

**Authors:** Patricia Torija, Alicia Robles, Ricardo Escalante

**Affiliations:** 1 Instituto de Investigaciones Biomédicas Alberto Sols. C.S.I.C./U.A.M., Calle Arturo Duperier 4, 28029 Madrid, Spain

## Abstract

**Background:**

Development of the post-genomic age in *Dictyostelium *will require the existence of rapid and reliable methods to disrupt genes that would allow the analysis of entire gene families and perhaps the possibility to undertake the complete knock-out analysis of all the protein-coding genes present in *Dictyostelium *genome.

**Results:**

Here we present an optimized protocol based on the previously described construction of gene disruption vectors by in vitro transposition. Our method allows a rapid selection of the construct by a simple PCR approach and subsequent sequencing. Disruption constructs were amplified by PCR and the products were directly transformed in *Dictyostelium *cells. The selection of homologous recombination events was also performed by PCR. We have constructed 41 disruption vectors to target genes of unknown function, highly conserved between *Dictyostelium *and human, but absent from the genomes of *S. cerevisiae *and *S. pombe*. 28 genes were successfully disrupted.

**Conclusion:**

This is the first step towards the understanding of the function of these conserved genes and exemplifies the easiness to undertake large-scale disruption analysis in *Dictyostelium*.

## Background

Comparative genomics is based on the conservation of the molecular function of genes in different organisms throughout evolution. Orthologous genes can be studied in simple, genetically tractable model systems, as a first step to address their function in higher organisms, including humans and evaluate their possible roles in diseases. The completion of *Dictyostelium *genome now offers the opportunity to study the function of conserved genes present in the social amoeba and other organisms in a systematic way [1].

*Dictyostelium *is a primitive eukaryote, living as a single cell organism while bacteria, its source of nutrients, are present in the soil. When bacteria are consumed, starvation triggers a complex response allowing the cells to aggregate by chemotaxis and form a multicellular structure. Many different aspects of its biology including motility, chemotaxis, cytokinesis, cell-differentiation and morphogenesis among others, are more closely related to those in higher organisms than to unicellular models, such as yeasts [2-4]. We have performed a systematic knock-out approach to begin to address the function of genes of unknown function present in *Dictyostelium *and human but absent from *S. cerevisiae *and *S. pombe *[5].

The classical approach of gene inactivation in *Dictyostelium *is performed by the insertion of a resistant cassette in the target gene by homologous recombination. Obtaining the disruption construct is time consuming since large flanking sequences are necessary to assure a high efficiency of the process. A PCR-based method and an *in vitro *transposition strategy have been developed facilitating the technique [6,7]. In the last method, the cloned gene is subjected to random insertion of a transposon containing a blasticidine-resistance cassette. The construct in which the transposon has interrupted the cloned gene is identified, expanded, digested and used for transformation of *Dictyostelium *cells. Since transposon insertion is random, it is sometimes necessary to screen a large number of clones, mainly when the gene is small. Besides, after transformation, the variable efficiency of homologous recombination in *Dictyostelium *makes the isolation of the disruptant strain tedious, since many different transformants must be screened to distinguish between random insertion and homologous recombination. Although all these difficulties are not a threat when few knock-outs are being performed, they can be overwhelming when we try to scale up the number of genes to study. We have therefore optimized all the steps from cloning of the gene, construction of the disruption vector, preparation of DNA for transformation and the screening of *Dictyostelium *transformants. We describe in detail the methods and illustrate its usefulness to disrupt a subset of genes of unknown function highly conserved between *Dictyostelium *and human.

## Results and discussion

### Optimizing the construction of disruption vectors in *Dictyostelium*

We have essentially followed the protocol described by [7] designed to insert a transposable cassette (EZTN:tetr-bsr), containing blasticidin- and tetracycline-resistance cassettes, into a cloned gene. The gene loci were previously amplified by PCR and cloned into pGEM-t vector as described in the methods section. Ideally, insertion events of the transposon must be in the middle of the clone, leaving large flanking regions to allow for an efficient homologous recombination. Besides, it might be important to interrupt the gene as much 5' as possible to disrupt the coded protein near the N-terminus. Since *in vitro *transposition is random, many different clones must be analyzed to find a correct location of the cassette. Consequently, the selection of the right insertion might be time-consuming, mainly when the targeted gene is small in comparison with the cloned insert. We have therefore designed a simple PCR strategy for a rapid assessment of the insertion point of the cassette after an *in vitro *transposition reaction. A single PCR reaction will provide information about the point of insertion and the same reaction can be sequenced directly to obtain the precise location of the insertion.

**Figure 1:**
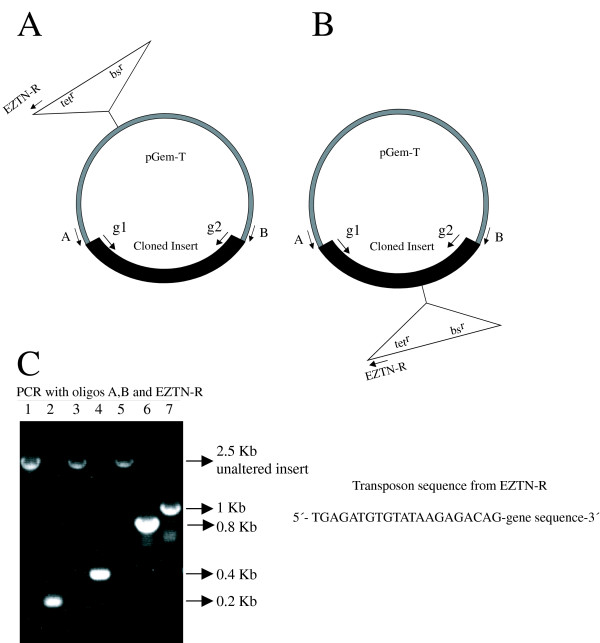
Optimizing the selection of the transposable cassette insertion into the target gene. A model of a cloned gene into the vector pGEM-t is shown in panels A and B. Two possible insertion events of the EZTN transposon are depicted: one in the vector (A) and the other in the cloned gene (B). The oligonucleotides used for cloning and analysis are shown as arrows. Panel C shows a PCR analysis of bacterial isolates after *in vitro *transposition. Oligonucleotides A, B and EZTN-R were used to estimate the approximate location of the transposon insertion. Lanes 1,3 and 5 correspond to insertion events probably located in the vector, as shown in the diagram of panel A. In this case, a 2.5 Kb insert corresponding to the size of the cloned gene is expected. Lanes 2,4,6 and 7 indicate insertion events in different points along the cloned gene, a situation illustrated in panel B. Panel C (right side) also shows the expected sequence obtained from the PCR products of lanes 2,4,6 and 7 by using the EZTN-R primer. The specific gene sequence located after the end of the transposon will indicate the precise location of the insertion as well as the orientation of the transposon.

Oligonucleotides A, B and EZTN-R (whose location is depicted in Figure 1) were used in a PCR reaction with DNA isolated from the bacterial colonies obtained after *in vitro *transposition as described in detail in the methods section. In those plasmids where the transposon was inserted in the vector, as illustrated in Fig 1A, a band corresponding to the size of the insert is expected (Fig 1C, lanes 1,3,5). However, if the insertion took place in the insert (see Fig 1B), EZTN-R oligo would be located in the right position for amplification with oligo A or B (depending on the orientation of the transposon). In this case, a band smaller than the size of the insert is expected, and its own size will provide an estimate of the position of the transposon (Fig 1C, lanes 2,4,6,7). In the case shown in Figure 1C, lanes 6 and 7 suggested good candidates. The specific insertion point can then be obtained by direct sequencing of the PCR product with oligo EZTN-R. The use of DNA extracted directly from the bacterial colonies and the procedure to obtained quality DNA for sequencing are optimized steps carefully described in the methods section.

### Analysis of gene disruption by PCR

**Figure 2:**
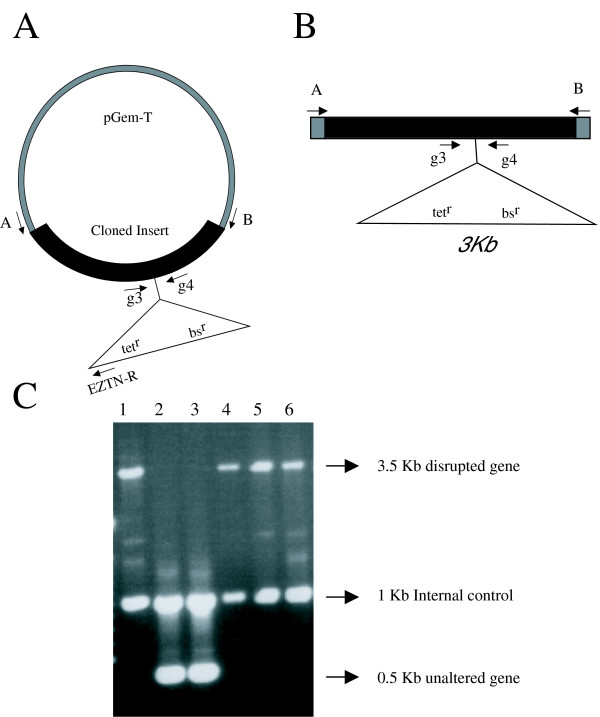
Rapid analysis of gene disruption by PCR. Panel A shows a diagram of a hypothetical disruption construct in which the cloned gene has been interrupted by the transposon. This construct was used as template for PCR with oligos A and B. The resulting fragment (depicted in panel B) was transfected into *Dictyostelium *cells. Panel C shows an example of the analysis of clonal isolates after transformation and selection with blasticidin. PCR with primers g3 and g4 generates a 0.5 Kb band, which is absent when the gene has been disrupted (lanes 1,4,5,6). The upper band confirms the insertion of the 3 Kb transposon in the locus.

The region containing the flanking sequences and the inserted transposon was amplified by PCR using the universal oligonucleotides A and B (see figure 2A,B for a schematic representation). Transformation of the PCR product is a faster alternative to the isolation of DNA by maxi-prep and subsequent digestion. Moreover, we have observed a higher transformation efficiency with PCR-derived DNA than that obtained with maxi-preps.

After selection, transformants were plated in association with *Klebsiella aerogenes *for clonal isolation. As soon as the lysis plaques are visible, cells from the growing zone were picked up and DNA extracted for PCR analysis. As depicted in Fig 2A,B, two close oligonucleotides surrounding the transposon insertion were previously synthethized for each targeted gene (g3, g4) to allow efficient amplification of the genomic region. A typical example of the analysis of *Dictyostelium *colonies by PCR is shown in figure 2C. Lanes 1,4,5 and 6 show the absence of the lower band corresponding to the amplification of the unaffected gene with oligos g3 and g4. Concomitantly, an upper band indicates the insertion of the transposon in the locus. An internal control corresponding to the amplification of an unrelated locus is included. The selected strains were grown and saved frozen for future analysis.

### Targeting genes of unknown function

In order to prove the value of the method, we have constructed 41 disruption constructs as described above for genes of unknown function present in *Dictyostelium *and human but absent from the genome of the yeasts *S. cerevisiae *and *S. pombe*. The level of homology of the putative proteins between *Dictyostelium *and human, as determined by E-value, was equal or lower than E-20. The disruption constructs were amplified by PCR and transformed in *Dictyostelium *by electroporation. 28 genes were successfully disrupted and for the remaining 13 we did not detect any homologous recombination events even after checking more than 100 independent transformants. Either the efficiency of homologous recombination in those loci was very low or alternatively, disruption of the gene leads to lethality. The data has been compiled in table 1 including those genes previously described (marked with an asterisk) [5]. Curated models of these predicted genes can be found at DictyBase [8]. The possible phenotype of the disruptants is now under investigation and will open the possibility to use *Dictyostelium *as a suitable model to address the function of these genes as previously described for MidA, a new mitochondrial protein involved in bioenergetics [5]. As expected, most of the genes are also represented in other model systems such as *Drosophila*, *Caenorhabditis *and *Arabidopsis*. However, a group of them are absent in some of the models. Remarkably, 6 of them (DDB0232143, DDB0217693, DDB0232153, DDB0217633, DDB0201847, DDB0187448) are present in human and *Dictyostelium *and no homologues can be recognized in the genomes of the mentioned model systems. In these particular cases, *Dictyostelium *is probably the only non-vertebrate model to study their function. The absence of these genes in the yeast models suggests that their function might be related with those aspects that are closer in *Dictyostelium *and higher eukaryotes than to unicellular protists. According to this, our preliminary phenotype analysis of the KO strains has revealed the involvement of some of these proteins in processes such as chemotaxis, motility, cytokinesis, phagocytosis and development (unpublished data).

## Conclusion

A complete protocol from gene cloning to isolation of disruptant strains in *Dictyostelium *is presented in this methodological report. Every step was optimized from gene cloning to selection of homologous recombination, with the aim to allow large-scale gene disruption strategies, such as the one described for the analysis of genes of unknown function. Once optimized, we have been able to complete the described protocol for a group of 10 genes in approximately 5 weeks of one person's work. It is conceivable for a well-trained technician to process 100 genes in a year. Extrapolating our results, we might expect to fail obtaining knock-out strains in around 30% of any group of genes, in part due to lethality. Other simple approaches must be designed to cope with these difficulties. Meanwhile, the existing methods, such as the one described here, will help to the rapid development of functional genomics in *Dictyostelium*.

## Methods

### Dictyostelium cell culture

Cells were grown axenically in HL5 medium (14 g/l tryptone, 7 g/l yeast extract, 0.35 g/l Na2HPO4, 1.2 g/l KH2PO4, 14 g/l glucose, pH 6.5) or in association with *Klebsiella aerogenes *in SM plates (10 g/l glucose, 1 g/l yeast extract, 10 g/l peptone, 1 g/l MgSO4·6H20, 1.9 g/l KH2PO4, 0.6 g/l K2HPO4, 20 g/l agar, pH 6.5) as described by [9].

### PCR-amplification and cloning of the genes of interest

Specific oligonucleotides were designed to amplify a genomic region of 2–3 Kb corresponding to the genes of interest. 1–2 μg of genomic DNA from AX4 was used as template in a standard 50 μl PCR reaction containing dNTPs at 0.5 mM each, oligonucleotides at 1 pmol/μl each, 1.5 units of Taq-polimerase (Biotools) and 1× PCR buffer from Biotools. Different cycle programs were used and the best results were obtained in the following ranges: initial melting: 5 min at 95°C; melting: 1 min at 95°C, annealing: 1 min at 45–50°C, elongation: 5–6 min at 62–65°C, 30 cycles; final elongation: 10 min at 62–65°C. The low extending temperature (62–65°C) allowed the amplification of highly A+T-rich templates as described previously [10]. 5 μl were used for checking the size and purity of the PCR product by agarose gel, the remaining of the reaction was purified with QIAquick PCR purification Kit from Qiagen and eluted in 30 μl. 3 μl were used for cloning into pGEM-T easy vector following manufacture's instructions. Ligation reactions were transformed into E. coli DH5α and the plasmids containing the inserts were recognized by restriction with NotI, which releases the inserted fragment. Plasmid DNA from alkaline mini-preps were purified with QIAquick PCR purification kit from Qiagen and eluted in 30 μl as described by manufacture's instructions. This purification step is essential for the efficiency of the in vitro transposition reaction described below, avoiding the necessity to perform maxi-preparation of the DNA.

### Insertion of the transposable cassette into the target gene

10 μg of the EZTN plasmid described by [7] were digested with PvuII and the reaction was directly purified with QIAquick PCR purification kit from Qiagen and eluted in 30 μl. We found that gel purification of the transposon was not necessary and increased the yield of the procedure. We used 1.5 μl of the pGEMT-cloned insert and 1 μl of the digested transposon in a reaction with 1 μl of transposase from Epicentre in a 5 μl final volume, following the manufacturer's instructions. After performing the transposition reaction and precipitation as described [7], E. coli DH5α were transformed and plated in LB-agar containing 15 μg/ml tetracycline, 50 μg/ml ampicillin. After 36 hours of incubation at 37°C, 10–20 bacterial colonies from each transposition were analyzed by PCR as described in figure 1. Bacterial colonies were picked up with a pipette tip, resuspended in 20 μl of distilled water and boiled for 5 minutes. 5 μl were used in a 50 μl PCR reaction with universal primers A and B, from pGEMt and EZTN-R oligo (5'-GCCAATATGCGAGAACACCCGAG-3'), derived from the sequence of the transposon [11]. The conditions for the PCR were as follows: 50 μl PCR reaction containing dNTPs at 0.25 mM each, oligonucleotides A, B and EZTN-R at 0.5 pmol/μl each, 1.5 units of Taq-polimerase (Biotools) and 1× PCR buffer from Biotools. The cycling program was as follows: initial melting: 5 min at 95°C; melting, 1 min at 95°C, annealing: 1 min at 50°C, elongation: 2 min at 65°C, 30 cycles; final elongation: 10 min at 65°C. 5 μl of the PCR reactions were used for analysis in agarose gels. The specific insertion point was obtained by direct sequencing of the PCR product with oligo EZTN-R. For this purpose, the remaining 45 μl of the PCR reactions were purified with QIAquick PCR purification kit from Qiagen and eluted in 30 μl. 3 μl were used for sequencing in an Applied Biosystems 377 sequencer. The expected sequence from oligo EZTN-R contains the transposase recognition sequence (which is indicated in Fig 1C), followed by the specific sequence of the targeted gene.

### PCR amplification of the whole construct and electroporation of *Dictyostelium *cells

Selected bacterial colonies containing the plasmid with the inserted transposon were grown overnight in LB supplemented with ampicilin and tetracycline. Alkaline mini-preps were performed and the plasmids digested with NotI for further confirmation. DNA from the mini-preps was purified with QIAquick PCR purification kit. We found this step essential for the quality of the DNA, necessary for PCR amplification in the subsequent steps. The region containing the flanking sequences and the inserted transposon was amplified by PCR using the universal oligonucleotides A and B (see figure 2A,B for a schematic representation). Four PCR reactions of 50 μl were set for each sample as follows: 1 μl of DNA from the purified mini-prep diluted to 1/50; dNTPs at 0.25 mM each, oligonucleotides A and B at 0.5 pmol/μl each, 2 units of Taq-polimerase (Biotools) and 1× PCR buffer from Biotools. Cycling program: initial melting: 5 min at 95°C, melting: 1 min at 95°C, annealing: 1 min at 55°C, elongation: 10 min at 62–65°C, 34 cycles; final elongation: 10 min at 62–65°C. The amplification product is expected to have 5.5–6 Kb (cloned insert plus transposon) and was checked by agarose electrophoresis. The four reactions were joined and purified with QIAquick PCR purification kit. DNA was eluted in 30 μl and 10 μl were electroporated in *Dictyostelium *cells as described by [12].

### Rapid analysis of gene disruption by PCR

After selection, transformants were plated in association with *Klebsiella aerogenes *for clonal isolation. As soon as the lysis plaques are visible, cells from the growing zone were picked up with a pipette tip and resuspended in 10 μl of MasterAmp DNA extraction solution from Epicentre. Samples were incubated 10 min at 60°C followed by other incubation of 10 min at 95°C. The DNA prepared in this way will be suitable for PCR amplification to screen for those transformants in which the gene has been disrupted by homologous recombination. 1 μl of the DNA was used in 50 μl PCR reaction containing 0.25 mM dNTPs, oligonucleotides g3, g4 and control oligos for amplification of an unrelated locus, at 0.5 pmol/μl each, 1.25 units of Taq-polimerase (Biotools) and 1× PCR buffer from Biotools. Cycling program: initial melting, 5 min at 95°C; melting: 1 min at 95°C, annealing: 1 min at 45°C, elongation: 3 min at 62°C, 30 cycles; final elongation: 10 min at 62°C. If necessary, this analysis can be performed in pools of clones to allow the rapid assessment of thousands of independent transformants as previously described [13].

## Authors' contributions

PT and AR designed and performed the experiments and also contributed to the manuscript. RE conceived the work, designed the experiment and wrote the manuscript.

## Figures and Tables

**Table 1:** Conserved genes of unkown function in Dictyostelium

Dicty gene (Dictybase ID)	Human gene (Genebank #)	Disruption vector (total aa/point of insertion)	Disrupted gene	
DDB0232140*	NP_653337	425/77	Yes	
DDB0232141*	CAH72141	164/135	Yes	
DDB0232143*	CAI14128	366/207	Yes	
DDB0232144*	AAH40291	715/233	Yes	
DDB0232146*	CAI12688	794/291	Yes	
DDB0232147*	BAA76798	1133/794	Yes	
DDB0232148*	AAY15088	260/92	Yes	
DDB0232149*	CAB64339	660/249	Yes	
DDB0232150*	BAB85063	930/375	Yes	
DDB0232151*	BAD96633	527/153	Yes	
DDB0232152*	BAA20779	2117/501	Yes	
DDB0232153*	BAA25518	1479/87	Yes	
DDB0232154*	BAD96633	567/183	Yes	
DDB0232155*	CAI22059	128/23	Yes	
DDB0232156*	NP_579877	688/327	Yes	
DDB0229859*	AAH04865	619/214	Yes	
DDB0187500	AAR27292	514/301	Yes	
DDB0218401	BAA13240	3080/2602	Yes	
DDB0187448	BAA92650	1099/407	Yes	
DDB0201847	BAA92650	1032/377	Yes	
DDB0202130	AAH94704	3779/388	Yes	
DDB0185464	NP_056090	1135/423	Yes	
DDB0217693	BAA25482	2322/1973	Yes	
DDB0188660	BAA20807	1550/614	Yes	
DDB0205660	AAH01079	183/61	Yes	
DDB0168698	CAH18422	315/64	Yes	
DDB0186376	CAB66646	403/35	Yes	
DDB0186924	BAB71412	675/430	Yes	
DDB0185679	AAH22898	195/47		No
DDB0185616	NP_056138	799/340		No
DDB0187996	BAD97348	1164/718		No
DDB0187107	BAB71377	978/344		No
DDB0185973	NP_115675	238/107		No
DDB0219747	BAA90931	676/274?		No
DDB0216747	CAG38656	1297/813		No
DDB0218704	Q8N201	2479/444		No
DDB0189962	AAI05095	1519/352		No
DDB0205483	AAH16707	508/301		No
DDB0186187	BAD96352	320/27		No
DDB0217633	NP_006484	322/150?		No
DDB0168130	CAB66565	385/183		No
				
41			28	13
